# Histopathological pattern of primary bone tumours and tumour-like lesions in Ile-Ife, Nigeria

**DOI:** 10.11604/pamj.2018.29.193.13111

**Published:** 2018-04-02

**Authors:** Obafemi Joel Aina, Kayode Adebowale Adelusola, Ayodele Elkanah Orimolade, Akinola Akinmade

**Affiliations:** 1 Morbid Anatomy Department, Federal Medical Centre Bida, Niger State, Nigeria; 2 Obafemi Awolowo University, Ile-Ife, Osun State, Nigeria

**Keywords:** Histopathological pattern, primary bone tumours, musculoskeletal tumours, tumour-like lesions

## Abstract

**Introduction:**

Bone tumours are relatively rare in comparison with neoplasms in other parts of the body. Previous studies have noted higher frequencies of these tumours in young adults with potentially devastating consequences.

**Methods:**

This study aimed to demonstrate the histopathological pattern of primary bone tumours and tumour-like lesions in Ile-Ife, Nigeria with emphasis on relative frequencies and distribution according to age, sex and anatomical location. A 13 year (1991-2003) retrospective study was carried out on 100 cases of primary bone tumours and tumour-like lesions. Records were retrieved from the surgical registers of the Histopathology Department, Qbafemi Awolowo University Teaching Hospitals Complex, Ile-Ife. Original histopathological slides were retrieved for examination and when they were unavailable, the paraffin blocks were searched out and new slides were made.

**Results:**

A total of 100 cases met the inclusion criteria for this study accounting for 1.55% of the 6,464 cases of all neoplasms seen within this period. Of the 100 cases, 50 were malignant (50%), 28 were benign (28%) and 22 had tumour-like lesions (22%). The male to female ratio was 1.3:1 and the most common benign and malignant tumours were osteochondroma and osteosarcoma respectively. The femur was the most frequently involved bone in primary malignant lesions (24%) followed by the pelvis and the maxilla (14% each).

**Conclusion:**

The age, sex and morphological distribution of benign and malignant bone tumours is similar to earlier reports in other African and international journals. Tumour-like lesions occur more frequently in females than in males. The femur is the most favourable site for malignant primary bone tumours and the jaw bones for tumour-like lesions.

## Introduction

Bone tumours are relatively rare in comparison with tumours in other parts of the body [1, 2]. Figures from the United States show an annual incidence of 2,100 cases of bone sarcoma. Considerable higher numbers were noted for lung cancer (93,000) and breast cancer (88,000) [1-4]. The precise incidence of primary bone tumours and tumour-like lesions is not known because many benign bone lesions are not biopsied for histopathological analysis [4]. These tumours present a challenge to the pathologist as well as the Orthopaedic surgeon. This challenge is magnified in an environment where resources are scarce. Late presentation also worsens outcomes and this is influenced by social, religious and other traditional beliefs among the population [5]. Bone tumours and tumour-like lesions have been found to occur mainly between the first and fourth decades of life. It is therefore clear that these tumours have a potentially devastating effect on the most productive segment of the population [6]. Oyemade et al and Omololu et al in their studies showed the possibility of geographical variations in the relative incidence of various histological types of bone tumours and their distributions according to age [7, 8]. Osteosarcomas have been found to peak in two age groups in England and Wales. The first peak occurs between the first and second decades of life coinciding with the pubertal growth spurt, while the second peak occurs after the fourth decade. However, such a second peak was not found in the studies done in Nigeria. This observation agrees with those made in Japan, the United States and Scandinavian countries and it has been attributed to a rarity of Paget's disease of the bone [7-9]. Mohammed et al in a 10 year retrospective review carried out in Zaria, Nigeria studied 127 patients with bone neoplasms. This number was 1.9% of the total 6,687 neoplasms reviewed during the period. Of those with bone neoplasms, 38.6% were benign tumours and 39.4% were malignant primary bone tumours. 22% were tumour-like lesions [10]. Obalum et al did a 25 year review of bone tumours in a tertiary health institution in Lagos, Nigeria and he found an annual average of about 100 cases. The most common benign and malignant tumours were osteochondroma and osteosarcoma respectively [6]. Variations have also been noted in the morphological distribution of these tumours. Earlier studies done in Nigeria have shown a high incidence of Burkitt lymphoma and a rarity of Ewing sarcoma, while studies in the United States and Asian countries showed the opposite [7,8, 11]. No study has been carried out within the locality of Ile-Ife on this subject hence the current thirteen year retrospective study. The aim is to determine the various histological types and relative frequencies of primary bone tumours seen in Obafemi Awolowo University Teaching Hospitals Complex, Ile-Ife. It is believed that this study will contribute to what is already documented on the epidemiology of primary bone tumours and tumour like conditions especially in Nigeria.

## Methods

A 13 year retrospective study was carried out covering the period between January 1^st^ 1991 and December 31^st^2003. Records of patients with a diagnosis of primary bone tumours and tumour-like conditions in the surgical registers of the Histopathological Department, Obafemi Awolowo University Teaching Hospitals Complex, Ile-Ife seen within the study period constituted the materials for this study. Original request cards, the patients' hospital records and X-ray films were retrieved for required information. The initial histological slides were also retrieved for examination. When such slides were faded or missing, the paraffin blocks were sought out and new slides were made. Tumours were classified as recommended by the WHO international reference centre for histological definition and classification of bone tumours. Histological criteria used in diagnosis and subtyping were also as recommended by the WHO [12]. Data was analyzed based on the histological types of tumours and their distribution according to age, sex and skeletal location using simple descriptive statistical methods.

## Results

A total of 6,464 cases of neoplastic conditions were diagnosed during the period of study. Of these, 2481 (38.4%) were benign and 3983 (61.6%) were malignant. 100 cases (1.55% of all cases seen) were selected for this study of which 57 were males and 43 were females giving a male to female ratio of 1.3:1 (Table 1). The average annual incidence of primary bone tumours during this period was 7.7%. Fifty cases (50%) were primary malignant bone tumours and the age range was 8 to 76 years with a mean age of 35.4 years and a peak incidence in the second decade of life. A summary of the age distribution of the various histological types of primary bone tumours and tumour-like lesions is shown in Table 2. Osteosarcoma was the most common primary malignant bone tumour seen, 21 cases (42%). The mean age for osteosarcomas was 21.33 years and majority (76.2%) were seen in the second and third decades of life. Nine cases occurred in the distal third of the femur (42.86%), three in the distal tibia (14.28%), three in the proximal tibia (14.28%), two in the mandible (9.52%) and one case each in the proximal third of the femur, the maxilla, humerus and calcaneus (4.76% each) as shown in Table 3. Twenty eight cases (28%) were benign and the age range was 4 to 60 years. Peak incidence was in the 3rd decade of life. Osteochondroma was the most common benign tumour seen and accounted for 32.1% of all benign bone tumours (9 cases). The tibia was the commonest site, contributing 33.3% of all cases (3 cases). Twenty two cases (22%) were tumour-like conditions. The age range was 9 to 65 years with a mean age of 31.5 years. Peak incidence was in the 2^nd^ and 3^rd^ decades of life (72.7%). The commonest site for tumour-like lesions was the maxilla as seen in 11 cases (50%) and the most frequent variant was fibrous dysplasia with 8 cases (36.4%).

**Table 1:** Sex distribution of primary bone tumours

Histological type	Male	Female	Total
**Malignant Tumours**			
Osteosarcoma	12	9	21
PCM	14	4	18
Chondrosarcoma	5	1	6
NHL	2	2	4
MGCT	-	1	1
**SUBTOTAL**	33	17	50
**Benign Tumours**			
Osteochondroma	6	3	9
Chondroma	4	4	8
Osteoma	-	1	1
BGCT	3	2	5
Myxoma	2	1	3
Fibromyxoma	-	2	2
**SUBTOTAL**	15	13	28
**Tumour-like lesions**			
Fibrous dysplasia	3	5	8
Ossifying Fibroma	6	5	11
CF	-	1	1
COF	-	1	1
MFD	-	1	1
**SUBTOTAL**	9	13	22
**OVERALL TOTAL (%)**	57	43	100

PCM – Plasma Cell Myeloma

NHL – Non-Hodgkin’s Lymphoma

MGCT – Malignant Giant Cell Tumour

BGCT – Benign Giant Cell Tumour

CF - Cementifying Fibroma

COF – Cemento-Ossifying Fibroma MFD – Metaphyseal Fibrous Defect (Non-Ossifying Fibroma)

**Table 2:** Age distribution of primary bone tumours

Histological type	0-10	11-20	21-30	31-40	41-50	51-60	61-70	> 70	Total
**Malignant Tumours**									
Osteosarcoma	2	8	8	1	2	-	-	-	21
PCM	-	-	1	4	2	6	4	1	18
Chondrosarcoma	-	5	-	1	-	-	-	-	6
NHL	-	-	-	1	1	-	2	-	4
MGCT	-	-	1	-	-	-	-	-	1
**Benign Tumours**									
Osteochondroma	3	2	2	1	1	-	-	-	9
Chondroma	2	1	4	-	1	-	-	-	8
Osteoma	-	-	-	-	-	1	-	-	1
BGCT	-	1	4	-	-	-	-	-	5
Myxoma	-	1	2	-	-	-	-	-	3
Fibromyxoma	-	1	-	1	-	-	-	-	2
**Tumour-like lesions**									
Fibrous Dysplasia	2	2	3	-	1	-	-	-	8
Ossifying Fibroma	-	4	5	2	-	-	-	-	11
CF	-	-	-	-	-	-	1	-	1
COF	-	1	-	-	-	-	-	-	1
MFD	-	-	1	-	-	-	-	-	1
**Total (%)**	9(9)	26(26)	31(31)	11(11)	8(8)	7(7)	7(7)	1(1)	100(100)

PCM - Plasma Cell Myeloma

NHL - Non-Hodgkin’s Lymphoma

MGCT - Malignant Giant Cell Tumour

BGCT - Benign Giant Cell Tumour

CF - Cementifying Fibroma

COF - Cemento-Ossifying Fibroma

MFD - Metaphyseal Fibrous Defect (Non-Ossifying Fibroma)

**Table 3:** Anatomical distributions of osteosarcomas

Anatomical site	Male	Female	Total	(%)
Upper third Femur	-	1	1	4.76
Lower third Femur	6	3	9	42.86
Upper third Tibia	2	1	3	14.29
Lower third Tibia	2	1	3	14.29
Mandible	1	1	2	9.52
Maxilla	-	1	1	4.76
Humerus	-	1	1	4.76
Calcaneus	1	-	1	4.76
**Total**	12	9	21	100

## Discussion

Bone tumours are comparatively rare globally and geographical variations in the occurrence of bone tumours have been established in many studies [1, 2, 6-11]. In this study, the malignant primary bone tumours accounted for 1.26% of the total malignant neoplastic lesions diagnosed over a period of 13 years (1991-2003). This finding is similar to that of Oyemade and Abioye in Ibadan who reported that primary malignant bone tumours accounted for 1.28% of all cancers [7]. This figure is however lower than that reported by Umar in Zaria (3.6%)), Kung;u in Kenya (2.5%) and Ahmad et al in Pakistan (3.14%). Lower rates were reported by Yeole and Jussawalla in Greater Bombay (0.9%) and Larsson et al in Sweden (0.7%) [13-15]. One hundred cases of primary bone tumours were identified within the study period giving an annual incidence of about 7.7 cases yearly. More cases were seen in a similar centre in Libya (16.5 per annum) and far more in the United States of America (3,000 per annum). The annual figures derived in Ile-Ife may not paint a true picture as most patients do not present to tertiary institutions due to cultural, religious and financial constraints. Indeed the annual incidence may be much higher. The relative frequencies of primary bone tumours in comparison with figures across the globe are shown in Table 4. Most cases in this study were malignant (50%) and this may be explained by the fact that many benign or supposedly benign lesions may not be sent for histopathological diagnosis as the threat to life is low unlike those thought to be malignant which the threat to life often lead to submission of samples for histopathological diagnosis. More males were affected with bone tumours with an overall male to female ratio of 1.3:1. This is very similar to the ratio of 1.25:1 reported by Odetayo in a similar study at Lagos [16]. For malignant primary bone tumours, the male to female ratio was 1.9:1. This is similar to the male to female ratio of 2:1 reported by Oyemade et al in a study of malignant primary bone tumours at Ibadan and Dahlin et al at Mayo clinic, USA. The ratio is however significantly different from 3.3:1 reported by Ahmad et al in Pakistan [7, 17, 18].

**Table 4:** Comparison of the relative ratio frequencies (rrf) of primary bone tumours in various studies

Study group	Total cases	Malignant	Benign	Tumour-like	Metastatic disease
Index study Ile-Ife 2006	100	50% (50)	28% (28)	22% (22)	_
Coard, West Indies 1998	136	39.7% (54)	50.7% (69)	9.6% (13)	_
Bahebeck et al Cameroon 2003	268	45% (121)	48% (129)	_	7% (18)
Sarma et al Libya 1994	165	24% (40)	_	76% (125)	_
Rao et al Karnataka India 1996	523	39% (204)	_	61% (319)	_
Solomon Johannesburg 1997	396	40.9% (162)	_	59% (234)	_

The peak age incidence for benign and malignant bone tumours was in the second and third decades of life (Table 2). This finding is in agreement with that of Oyemade (Ibadan), Omololu et al (Ibadan), Odetayo (Lagos) and Solomon (South Africa) [7, 8, 16, 19]. However, the report by Umar in Zaria is slightly different; the peak age incidence in Umar's work was in the first and second decade of life [20]. This was because Umar's work contained many cases of Burkitt lymphoma which was more frequent in the 1^st^ decade of life. No single case was seen in this present study. This was because in Obafemi Awolowo University Teaching Hospital Complex, Ile-Ife, most cases of Burkitt lymphoma are diagnosed through fine needle aspiration cytology (FNAC). Tissue biopsy for histological diagnosis of Burkitt lymphoma is rarely done. Only cases with tissue biopsy histological diagnosis were included in this study. For benign tumours, the peak age incidence was in the 3^rd^decade of life, during which 12 cases (42.9%) out of the total 28 cases were seen. This finding is similar to that of Umar at Zaria, who reported that 70 cases (36.6%) out of 191 cases of benign tumours were seen in the third decade of life. The third decade peak was also true for tumour-like lesions in this present study. However, the study by Solomon in South Africa showed a 2^nd^ decade incidence peak for benign tumours and tumour like lesions [19]. The most frequently involved bone in primary malignant bone tumours, in this study, was the femur, which was involved in 12 cases (24%). This finding was consistent with that of Solomon in South Africa and Ahmad et al in Pakistan [18, 19]. They both reported femur as the most frequently involved bone in primary malignant tumours. However, Omololu et al at Ibadan reported that the mandible was the most frequently involved bone in primary bone cancer, followed by the femur, tibia and maxilla in decreasing order [8]. For tumour-like lesions, the maxilla and the mandible were the most frequently involved. This is similar to report by Umar in Zaria, which showed that the mandible and the maxilla were also more affected by tumour-like lesions [20]. The relative ratio frequencies of various histological types of primary bone tumours and the distribution of each histological type in relation to age, sex and skeletal location share similar patterns with those reported in other studies (Table 1, Table 2, Table 3).

Osteosarcoma was the commonest malignant bone tumour in this study. It accounted for 42% of all bone cancers. This figure of relative frequency ratio is very close to 39% reported by Bahebeck et al in Cameroon and Umar in Zaria, 45.7% by Rao el at in India and 36.0% by Oyemade et al at Ibadan [7, 21, 22]. The figure is however significantly lower than 66% reported by Odetayo in Lagos and 60.7% by Omololu et al in Ibadan [8]. The figure of 28.8% reported for Sweden is significantly lower than 42% in this study. There was a preponderance of Osteosarcoma in males with a male to female ratio of 1.3:1 in this study. This correlates well with reports of other investigators in Ibadan, Nigeria, other African countries, India, Pakistan Europe and America. The high incidence of this tumour in the second and third decades of life (76.2%) coincides with the pubertal growth spurt. Histopathological analysis in this study showed that the conventional (high grade) pattern was the most predominant type seen (76%) for Osteosarcoma. This is in agreement with reports from studies in other centres. Four cases of Non-Hodgkin lymphoma were diagnosed in this study representing 8% of all primary bone tumours. This is similar to 10.6% reported by Shah et al in Pakistan [23]. The predominant histological variant of Non-Hodgkin lymphoma was diffuse large cell type (50%). Of the benign lesions, osteochondroma was the most common (32.1%). Kung'u, Umar and Dahlin reported frequencies of 32.6%, 30% and 35.8% respectively [14, 17, 20]. Male to female ratio in this study was 2:1. These figures as well as the age and skeletal distribution are consistent with those reported by other studies. Ossifying fibroma, cementifying fibroma and cemento-ossifying fibroma are closely related lesions which accounted for 59.2% of the tumour-like lesions in this study. They were found to occur predominantly in the second and third decades of life and affected only the jaw. Umar's report however showed more cases affecting the tibia and phalanges [20]. This study further contributes to what is already known regarding the histopathological patterns and the demographics of primary bone tumours and tumour-like lesions in our environment. Limitations encountered include the study design being retrospective with attendant problems of data gathering as well as our inability to carry out further high end histopathological analysis due to dearth of requisite technology.

## Conclusion

Bone tumours are comparatively rare. This study showed that benign and malignant primary bone tumours are more common in males than in females while tumour-like lesions affect more females than males. The femur was found to be the most favourable site for primary malignant bone tumours while the jaw bones were most commonly affected by tumour-like lesions. The distribution of primary bone tumours and tumour-like lesions, with respect to age, sex and anatomical sites, shows striking similarity with that seen in many other parts of the world.

### What is known about this topic

- Epidemiological figures for bone tumours and tumour-like lesions exist for several parts of Nigeria, West Africa and the World generally;
- Several geographical variations have been noted with regard to the manner of presentation, anatomical site affected and frequency of occurrence.

### What this study adds

- Figures are now available for the locality of Ile-Ife, Osun state and will serve as a template upon which future studies in this field can be built;
- This study also provides the histopathological pattern of tumours and tumour-like lesions which is not widely studied;
- We recognize the fact that the data is not very current however we the authors feel the need to publish these findings for future reference.

## Competing interests

The authors declare no competing interests.
